# Population-Based Study of Prevalence, Bother and Behavior Related to Treatment for Lower Urinary Tract Symptoms and Overactive Bladder among Polish Neurogenic Patients

**DOI:** 10.3390/brainsci11060712

**Published:** 2021-05-27

**Authors:** Mikolaj Przydacz, Marcin Chlosta, Tomasz Golabek, Piotr Chlosta

**Affiliations:** Department of Urology, Jagiellonian University Medical College, ul. Jakubowskiego 2, 30-688 Krakow, Poland; marcin.p.chlosta@gmail.com (M.C.); elementare@op.pl (T.G.); piotr.chlosta@gmail.com (P.C.)

**Keywords:** Poland, epidemiology, neurology, neurogenic bladder, neurogenic lower urinary tract dysfunction

## Abstract

Background: The aim of this study was to perform a cross-sectional study of Polish neurogenic patients to measure, at the population level, the prevalence, bother and behavior associated with treatment for lower urinary tract symptoms (LUTS) and overactive bladder (OAB). Methods: This epidemiological study was based on data from LUTS POLAND, a computer-assisted and population-representative telephone survey. Participants were classified by age, sex and place of residence. Results: LUTS POLAND includes 6005 completed interviews, of which 1166 (19.4%) were for individuals who had ever received any treatment by neurologists and/or neurosurgeons. Among these neurogenic participants, LUTS prevalence was 72.3%, statistically higher than for non-neurogenic respondents. At the population level, neurogenic patients had about a 20% higher risk for LUTS presence than non-neurogenic participants (relative risk: 1.17–1.21). LUTS prevalence did not differ between men and women. Frequency was the most common of the LUTS. Forty percent of neurogenic respondents described having more than one LUTS subtype (i.e., storage, voiding, and/or post-micturition symptom subtype), and more than 50% of respondents reported OAB symptoms. Both storage and voiding symptoms were bothersome, and many neurogenic individuals (42.3–51.0%) expressed anxiety about bladder function affecting quality of life. Only one-third (34.9–36.6%) of neurogenic participants had sought treatment for their LUTS, and the majority of such individuals received and maintained treatment. Conclusions: LUTS and OAB symptoms were highly prevalent and bothersome among Polish neurogenic patients at the population level. Because the scale of seeking treatment for LUTS was low, Polish neurogenic patients may not be adequately informed about multiple effects of LUTS and OAB.

## 1. Introduction

Adult neurogenic lower urinary tract dysfunction (ANLUTD) due to central and/or peripheral nervous system disease significantly deteriorates quality of life and survival [1]. Disorders or injuries such as stroke, multiple sclerosis, Parkinson’s disease and spinal cord injury may lead to abnormal functioning of the bladder–urethra complex [2]. Notably, the type of urological dysfunction varies with the type and degree of damage to the central or peripheral nervous system. The bladder can become underactive or overactive, with urethral complex deficiency or detrusor-sphincter dyssynergia [3]. Therefore, ANLUTD patients report a large variety of lower urinary tract symptoms (LUTS) that includes storage symptoms (with overactive bladder (OAB) syndrome), voiding symptoms, and post-micturition symptoms.

Currently, there are minimal population-level data on overall prevalence, bother and behavior related to treatment for LUTS and OAB symptoms among neurogenic patients. There is, in particular, a dearth of information extracted from representative groups. Indeed, the European Association of Urology Guidelines on Neuro-urology highlight the lack of overall population estimates [4]. To date, no study in any country has evaluated neuro-urological patients at the population level using reliable definitions provided by the International Continence Society (ICS), or with adequate instruments [1,5]. The ICS specifies that investigators should adhere to widely accepted definitions to conduct high-quality research [1]. Further, whereas some epidemiological data, mainly from central registers and concentrated on treatment-related behaviors, are available from some countries of Western Europe and North America, little epidemiological data exist for other regions of the world, including Eastern Europe [6]. Because multiple aspects may affect health-related behavior, treatment behavior related to urological symptoms in neurogenic patients in Poland or other Eastern European countries may vary. Data regarding treatment behavior are necessary to foster health, increase awareness, and minimize the toll of disease because ANLUTD, apart from exerting a negative impact on quality of life, may lead to severe complications (e.g., renal deterioration, urinary tract infections and urolithiasis) if not managed and monitored effectively. Population estimates expedite the formation of interdisciplinary foundations for national health programs, and they promote the adequate allocation of government and healthcare system resources [7]. Therefore, the aim of this analysis was to investigate, at the population level, the overall prevalence, bother and behavior related to treatment for LUTS and OAB symptoms in a group of Polish neurogenic patients.

## 2. Methods

The investigation described in this report focuses on data from a subset of individuals, i.e., neurogenic persons, taken from LUTS POLAND, our cross-sectional, population-representative survey of LUTS and OAB in Poland. Detailed descriptions of the concepts, study design, methodology, and data collection for LUTS POLAND are published and reported only in brief in this paper [7,8]. LUTS POLAND surveyed men and women, aged ≥40 years, who were living across all of Poland, in urban and rural areas. The Jagiellonian University Medical College Ethics Committee approved this study (1072.6120.160.2019). In addition, the study was registered with ClinicalTrials.gov (NCT04121936).

### 2.1. Study Design and Data Collection

We designed a target sample based on the latest census and a sample matching technique [9,10,11]. Ipsos Poland conducted data collection [12]. All participants were queried about demographics and the presence of LUTS, as specified by the ICS. LUTS included storage symptoms, voiding symptoms, and post-micturition symptoms [5]. Participants rated the occurrence of all symptoms during the prior month. Participants used a Likert-like scale to rate symptom occurrence (none, less than 1 in 5 times, less than half the time, about half the time, more than half the time, almost always). Additionally, respondents rated the bother that accompanied the symptoms (not at all, a little bit, somewhat, quite a bit, a great deal, a very great deal). In addition, we included questions from the International Prostate Symptom Score (IPSS) [13] and the Overactive Bladder-Validated 8-question Screener (OAB-V8) [14]. During the interview, participants also assessed how bladder problems affected their seeking and receiving of treatment, satisfaction with their treatment, treatment continuation, and quality of life. Lastly, respondents provided information about whether they had received treatment generally from any type of healthcare professional and, if yes, by what type of specialist. The analyses in this report focus only on data of neurogenic respondents, i.e., those who had ever received any form of treatment by neurologists and/or neurosurgeons.

### 2.2. Objectives

The primary study objective was to investigate the prevalence of LUTS in a subpopulation of persons who had ever received any form of treatment by neurologists and/or neurosurgeons (i.e., in a subpopulation of neurogenic patients). Researchers who conducted large-scale international studies of the general population have used either of two definitions for LUTS prevalence [7,15,16]. To be able to compare our findings with findings from those earlier epidemiological analyses, we used both definitions of LUTS prevalence: definition I, symptoms occurring “less than half the time” or more, and definition II, symptoms occurring “about half the time” or more. This methodology enabled us to accurately juxtapose the data exclusively for individuals treated by neurologists and/or neurosurgeons with the data from other analyses, typically conducted in the general population. We pre-specified the primary objective for this analysis in the statistical analysis plan, before initiating the survey.

The secondary study objectives were related to documenting the prevalence of specific LUTS, the bother of specific LUTS (LUTS rated at least “quite a bit” were considered bothersome), the OAB prevalence (based on the score ≥8 points from the OAB-V8), quality of life (based on the IPSS), and behavior related to the treatment for LUTS.

### 2.3. Statistics

Descriptive statistics were used for demographic variables and initial data evaluation. Continuous (numeric) variables were subjected to the Kruskal–Wallis test, and categorical variables (containing a finite number of categories or distinct groups) were analyzed by the chi-squared test to evaluate differences in LUTS prevalence. Relative risks were calculated as the prevalence rate of an outcome in the exposed group, (i.e., neurogenic respondents), divided by the outcome of the unexposed group (i.e., non-neurogenic respondents). In addition, we used the logistic regression model to measure differences in LUTS/OAB prevalence regardless of age (a signature risk factor for LUTS) [17]. A *p* value <0.05 was considered statistically significant. We used SPSS Statistics software (IBM, version 24.0) for all data analyses.

## 3. Results

The LUTS POLAND survey included 6005 respondents from throughout Poland, and 1166 (19.4%) of these persons reported to have been treated by neurologists and/or neurosurgeons. We identified more women (*n* = 695; mean age 63 ± 11.1) than men (*n* = 471; mean age 63 ± 11.8) in the neurogenic cohort. Notably, the neurogenic individuals had a mean age of 63, slightly higher than the mean age of 60 for the non-neurogenic respondents (*p* < 0.01). More neurogenic participants resided in urban (*n* = 723) than in rural areas (*n* = 443). 

### 3.1. The Prevalence of LUTS

In our neurogenic cohort, the prevalence of at least one LUTS at least “less than half the time” (definition I) was 72.3% (*n* = 843); women (*n* = 503; 72.4%) and men (*n* = 340; 72.2%) exhibited the same prevalence. According to definition II, at least one LUTS at least “about half the time”, the prevalence was 65.4% (*n* = 762), again with no difference between women (*n* = 454; 65.3%) and men (*n* = 308; 65.4%). By both definitions, LUTS were more prevalent in neurogenic than non-neurogenic participants (definition I: 72.3% vs. 59.5%; definition II: 65.4% vs. 52.0%; *p* < 0.01). Further, the higher prevalence of LUTS in neurogenic persons was independent of age (definition I: relative risk 1.17, 95% confidence interval 1.06–1.31, *p* < 0.01; definition II: relative risk 1.21, 95% confidence interval 1.09–1.41, *p* < 0.01). We did not observe urban versus rural differences in LUTS prevalence.

### 3.2. The Prevalence of Specific LUTS

Frequency followed by nocturia and urgency were the most prevalent symptoms overall in the group of neurogenic patients (Table 1).

Among storage symptoms, frequency, nocturia, urgency, urgency with fear of leaking, urgency urinary incontinence, stress urinary incontinence, and leak for no reason, were more prevalent in neurogenic vs. non-neurogenic participants (Table 1).

For voiding and post-micturition symptoms, intermittency, slow stream, hesitancy, straining, and incomplete emptying, were more prevalent in neurogenic vs. non-neurogenic respondents (Table 1).

LUTS has a strong age dependency. Thus, we performed an age-adjusted analysis and found that, regardless of age (*p* < 0.01), frequency, nocturia, urgency, urgency urinary incontinence, leak for no reason, and straining, were still more prevalent in neurogenic vs. non-neurogenic respondents.

### 3.3. The Prevalence of LUTS Subgroups

In the group of neurogenic patients, storage symptoms comprised the most prevalent ICS symptom group (definition I: 69.2%; definition II: 62.9%). Neurogenic participants reported voiding symptoms of 33.2% and 22.4% according to definition I and definition II, respectively. Post-micturition symptoms were the least prevalent (definition I: 17.6%; definition II: 10.1%). Notably, 44.1% of neurogenic patients reported having LUTS from two (or more) different symptom subtype groups, as defined by ICS.

### 3.4. The Bother of Specific LUTS

Among the neurogenic patients, the most bothersome symptoms were urgency with fear of leaking, leak for no reason, and straining (Table 1). Interestingly, whereas the bother from all storage symptoms was comparable between neurogenic and non-neurogenic participants, the bother from all voiding symptoms was greater in neurogenic individuals compared with non-neurogenic respondents.

### 3.5. The Prevalence of OAB

OAB symptoms, evaluated with the OAB-V8 questionnaire (i.e., score ≥8 points), had a 53.5% prevalence among the neurogenic individuals, considerably higher than the 37.9% prevalence in the non-neurogenic group (*p* < 0.01). Importantly, the higher prevalence of OAB in the neurogenic group was independent of age (*p* < 0.001).

### 3.6. The Effect of LUTS on Quality of Life

With the question: “If you were to spend the rest of your life with your urinary condition just the way it is now, how would you feel about that?”, we found that LUTS impaired quality of life. Based on definition I, 42.3% of the neurogenic patients with LUTS responded with “mixed”, “mostly dissatisfied”, “unhappy”, or “terrible”. Using definition II, 51.0% of neurogenic patients with LUTS responded similarly.

### 3.7. Treatment Seeking and Treatment Receiving

Based on definition I, one-third (34.9%, *n* = 294) of neurogenic individuals with LUTS sought LUTS treatment, and most received treatment (32.3%, *n* = 272). With the definition II criterion, 36.6% (*n* = 279) of neurogenic respondents pursued LUTS treatment and most obtained treatment (35.2%, *n* = 268). We did not identify disparities between treatment seeking/receiving and urban/rural areas.

### 3.8. Treatment Satisfaction and Treatment Continuation

More than half of the neurogenic patients who underwent treatment for LUTS maintained treatment (definition I: 61.0%; definition II: 63.3%). However, less than half of neurogenic respondents were satisfied with their therapy (definition I: 46.3%; definition II: 45.2%). We did not identify differences between status of treatment continuation/discontinuation, treatment satisfaction/dissatisfaction, and urban/rural areas.

## 4. Discussion

The assessments in this investigation are an extension of the LUTS POLAND study that examined all of Poland, including adequate proportions of both urban and rural regions. Importantly, this analysis is also the first in the literature of LUTS and OAB symptoms among neurogenic patients that provides overall population estimates based on a single-country, representative group of adults. In addition, this study is the first in Eastern Europe that analyzed, at the population level, the treatment-related behavior for LUTS and OAB symptoms in neurogenic patients. Our study corroborates the recommendations of the European Association of Urology, which strongly advocates inclusion of the general population in studies that analyze correlations between LUTS/OAB and neurogenic diseases [4]. The immense effect of ANLUTD on public health has been brought into focus by the substantial improvement in the understanding of different clinical aspects of urological consequences following neurogenic diseases, a topic of increasing interest [18].

The prevalence of LUTS at the population level has been estimated from a cluster of large international epidemiological studies [15,16]. However, all these earlier studies examined the general population with no separate and further analyses of individuals treated by healthcare professionals with different specialties. We found that LUTS were highly prevalent specifically in a large cohort of neurogenic patients at the population level compared with the prevalence of LUTS in the group of participants without neurogenic conditions. Notably, at the population level, neurogenic patients had about a 20% higher risk for LUTS presence compared with non-neurogenic participants regardless of age (relative risks: 1.17–1.21). Interestingly, in our study, neurogenic men and women did not differ in LUTS prevalence, although most studies of the general population showed that LUTS were more prevalent in women than in men [7,15,16]. The absence of a difference by sex for neurogenic individuals substantiates the concept that LUTS induced by neurogenic conditions occur by their own specific, sex-independent mechanisms [19].

Despite the fact that storage symptoms were more prevalent than voiding or post-micturition symptoms, the coexistence of multiple and different symptoms, from different ICS symptom groups/categories, was common and occurred in more than 40% of neurogenic respondents. This finding is particularly noteworthy because the urological dysfunction in neurogenic patients depends on the extent and duration of each specific disorder [3]. Clinical presentation of ANLUTD may vary greatly between different neurogenic disorders with combinations of multiple symptoms that often result from combined bladder–sphincter dysfunctions. Further, evolution of bladder–sphincter behavior is often observed with the progression of a primary disease (e.g., multiple sclerosis or Parkinson’s disease) [2]. Moreover, because some neurogenic disorders have become lifelong conditions (e.g., spinal cord injury), increased survival exposes patients to different neurological and urological changes that modify bladder–sphincter behavior [18]. Therefore, neuro-urological patients always require extensive and thorough diagnostic LUTS evaluation.

Special consideration should be given to frequency, a complaint that an individual voids too often by day [1,5]. Importantly, frequency was the most prevalent symptom in the neurogenic respondents, whereas the most prevalent symptom in the group of non-neurogenic participants was nocturia. Further, the OAB-V8 questionnaire, a screening tool for OAB symptoms, revealed that more than half of our neurogenic respondents surpassed the threshold for clinically significant OAB symptoms, statistically more than non-neurogenic participants. In neurogenic patients, frequency and other OAB symptoms commonly correlate with neurogenic detrusor overactivity. Neurogenic detrusor overactivity occurs in a wide range of neurogenic conditions, including suprapontine and infrapontine-suprasacral lesions, when cortical inhibition of voiding reflex is disturbed [2]. Therefore, our results support the differences in prevalence of LUTS between different populations, along with the underlying inference that data from the general population should not be extrapolated to different subpopulations of patients [20].

LUTS can be highly bothersome and degrade the quality of life. In the general population, storage symptoms were found to be more bothersome than voiding or post-micturition symptoms [7,15,16]. However, for neurogenic respondents, storage symptoms were characterized by comparable levels of bother to voiding symptoms. This finding may highlight the fact that mechanisms evoking voiding symptoms in neurogenic patients, mainly detrusor-sphincter dyssynergia and detrusor underactivity, affect patient perception of bother more so than mechanisms leading to voiding symptoms in non-neurogenic individuals.

Only one-third of neurogenic patients with LUTS were seeking treatment, with no differences between urban and rural areas. Multiple reasons have been proposed for the variability in rates of treatment seeking by persons with LUTS in the general population. However, neurogenic individuals require a high level of care; it is of utmost importance to adequately diagnose and regularly follow these patients to avoid life-threatening complications and preserve quality of life (continence and sexual function) [18]. Although there are extensive guidelines and recommendations exclusively dedicated for neuro-urological patients [4], investigators report that, even in developed countries, patients with ANLUTD are often not diagnosed and monitored appropriately [6,21,22]. Thus, improvement of patient awareness of the nature of ANLUTD is a persistent need. As treatment for ANLUTD is of great importance, the lack of recognition of the need for treatment and knowledge about treatment options may present barriers to healthcare seeking. Importantly, education, counselling and increased awareness of ANLUTD can be provided not only by urologists but also by different types of clinicians. Fortunately, the importance of urology care in neurogenic patients has gained attention in Poland and other Eastern European regions [23,24]. Future health improvement programs in Poland need to definitively reach neuro-urological patients.

This study had limitations, which were mainly related to the nature of the data capture and data self-reporting. Importantly, in routine clinical practice, ANLUTD can be diagnosed in patients with a relevant neurological condition. However, we did not ask participants about specific neurogenic disorders. During a telephone interview, and without clinical verification, it would have been difficult to obtain reliable information about specific disorders from a self-reporting participant [25]. In addition, it must be remembered that, for neurogenic patients, a urodynamic study remains a basic investigation because it is crucial in clinical decision-making [4]. However, the application of urodynamics for studies at the population level is extremely restricted. We limited our survey to a pool of adults aged ≥40 years; therefore, younger representatives were not addressed. Nonetheless, most large and international population-based investigations of LUTS and OAB included respondents of ≥40 years of age [15,16]. The survey methodology was designed such that we could broadly compare our results with other populations. In addition, most neurology disorders that lead to ANLUTD appear after 40 years of age, even spinal cord injuries [26]. We also need to admit that our cohort included participants who had ever received any treatment by neurologists or neurosurgeons. Thus, our sample may not be highly neurologically homogenous. With this approach, our population, for instance, included those continuously treated by neurologists/neurosurgeons, as well as those who might have consulted with neurologists/neurosurgeons only once during their lifespan. Nonetheless, with population-representative studies, certain estimates and parameters require generalization (e.g., merging data). With our study, we needed to provide results for the whole population in order to produce estimates that are capable of improving national health programs through the adequate allocation of resources by governments and healthcare systems. In addition, we need to admit that investigation of the effect of LUTS on quality of life with only one question may not fully reveal the relationship between LUTS and quality of life. However, we limited the number of questions in our survey to foster respondent compliance. We did not ask all possible questions (including those from multiple questionnaires) so as not to overwhelm the respondents. Finally, some of the population estimates, particularly behaviors related to treatment, may not be universally generalizable and rigidly refer to other populations because cultural norms may restrain individuals from discussing their health issues [27].

In conclusion, this study is the first population-based investigation of a large cohort of neurogenic patients among a larger representative pool of adults for which an analysis was performed for the overall prevalence, bother and behavior related to treatment for LUTS and OAB symptoms. Neurogenic patients exhibited a relatively high prevalence of LUTS, higher than the prevalence among non-neurogenic respondents. The coexistence of multiple symptoms was often observed. LUTS were frequently bothersome and impaired quality life. However, relatively few individuals sought treatment for their LUTS. Therefore, we need to develop strategies to specifically increase the population awareness about the multiple negative effects of ANLUTD on patient quality of life and overall survival, while optimizing cooperation between professionals with diverse healthcare specialties.

## Figures and Tables

**Table 1:** Prevalence of specific symptoms according to definition I (symptoms occurring less than half the time or more) and definition II (symptoms occurring about half the time or more) and associated bother in neurogenic and non-neurogenic participants.

	Neurogenic Participants (*n* = 1166)						Non-Neurogenic Participants (*n* = 4839)					
	Symptom Prevalence (Definition I)		Symptom Prevalence (Definition II)		Prevalence of Bother (At Least Quite a Bit) ^a^		Symptom Prevalence (Definition I)		Symptom Prevalence (Definition II)		Prevalence of Bother (At Least Quite a Bit) ^a^	
	*n*	%	*n*	%	*n*	%	*n*	%	*n*	%	*n*	%
Storage symptoms												
Nocturia ^b^	497	42.6 **	285	24.4 **	156	54.7	1600	33.1	683	14.1	365	53.4
Frequency	512	43.9 **	346	29.7 **	196	56.7	1395	28.8	929	19.2	423	45.5
Urgency	315	27.0 *	199	17.1 *	168	84.4	856	17.7	474	9.8	385	81.2
Urgency with fear of leaking	271	23.2 *	182	15.6	173	95.1	720	14.9	409	8.5	377	92.2
Urge urinary incontinence	139	11.9 *	71	6.1	65	91.5	286	5.9	171	3.5	144	84.2
Stress urinary incontinence	144	12.3 *	86	7.4	77	89.5	324	6.7	193	4.0	180	93.3
Mixed urinary incontinence ^c^	105	9.0	55	4.7	49	89.1	275	5.7	168	3.5	153	91.1
Leak for no reason	79	6.8 *	45	3.9 *	41	91.1	148	3.1	78	1.6	68	87.2
Voiding symptoms												
Intermittency	177	15.2 *	120	10.3 *	90	75.0 *	362	7.5	206	4.3	98	47.6
Slow stream	218	18.7	135	11.6 *	97	71.9 *	561	11.5	299	6.2	117	39.1
Hesitancy	134	11.5 *	74	6.3 *	59	79.7 *	307	6.3	139	2.9	68	48.9
Straining	88	7.5 *	49	4.2 *	46	93.9 *	180	3.7	91	1.9	47	51.6
Splitting/ spraying	115	9.9	68	5.8	55	80.1 *	312	6.4	144	3.0	60	41.7
Terminal dribble	239	20.5	150	12.9	96	64.0 *	657	13.6	396	8.2	149	37.6
Post-micturition symptoms												
Incomplete emptying	170	14.6	105	9.0 *	83	79.0 *	406	8.4	222	4.6	126	56.8
Post-micturition dribble	87	7.5	38	3.3	30	78.9	219	4.5	117	2.4	97	82.9

^a^ Prevalence of bother was based on definition II. ^b^ Nocturia was defined as two or more voids per night. ^c^ Participants who reported both urge and stress urinary incontinence symptoms were classified as having mixed urinary incontinence. * *p* ≤ 0.05, neurogenic vs. non-neurogenic participants. ** *p* ≤ 0.01, neurogenic vs. non-neurogenic participants.

## Data Availability

Data is contained within the article.
